# Development of a γ-Cyclodextrin-Based Cryogel Loaded with Trimethoprim for Acne Treatment: Design, Synthesis, and In Vitro Evaluation

**DOI:** 10.3390/ijms26136319

**Published:** 2025-06-30

**Authors:** Elisabetta Grazia Tomarchio, Valentina Giglio, Virginia Fuochi, Salvatore Furnari, Pio Maria Furneri, Tommaso Mecca, Sandro Dattilo, Chiara Zagni, Antonio Rescifina

**Affiliations:** 1 Department of Drug and Health Sciences, University of Catania, V.le A. Doria 6, 95125 Catania, Italy; elisabetta.tomarchio@phd.unict.it (E.G.T.); antonio.rescifina@unict.it (A.R.); 2 Department of Biomedical and Biotechnological Sciences, University of Catania, Via Santa Sofia 97, 95123 Catania, Italy; virginia.fuochi@unict.it (V.F.); salvatore.furnari@phd.unict.it (S.F.); furneri@unict.it (P.M.F.); 3 Institute of Biomolecular Chemistry CNR-ICB, Via Paolo Gaifami 18, 95126 Catania, Italy; valentina.giglio@cnr.it (V.G.); tommaso.mecca@cnr.it (T.M.); 4 Institute for Polymers, Composites, and Biomaterials CNR-IPCB, Via Paolo Gaifami 18, 95126 Catania, Italy; sandro.dattilo@cnr.it; 5 Consorzio Interuniversitario Nazionale Metodologie e Processi Innovativi di Sintesi (CINMPIS), Via E. Orabona 4, 70125 Bari, Italy

**Keywords:** cryogel, γ-cyclodextrin, trimethoprim, inclusion complex, topical drug delivery, acne treatment, sustained release, antibacterial

## Abstract

Innovative functional materials integrating host–guest complexes in cryogels offer promising applications in topical drug delivery, enhancing drug solubility and stability. In this study, we designed and developed a cryogel-based patch for acne treatment by polymerizing an acrylate-functionalized γ-cyclodextrin (γ-CD) and trimethoprim (TMP) inclusion complex with [2-(acryloyloxy)ethyl]trimethylammonium chloride (AETMA) at low temperatures. A multistep workflow was applied to synthesize the inclusion complex via mortar-assisted kneading, followed by cryogel formulation through radical cryopolymerization. The resulting hybrid system leverages the cationic nature of AETMA to promote adhesion and electrostatic interactions with the skin surface. At the same time, γ-CD serves as a drug reservoir, facilitating sustained release of the drug. The system was characterized by FT-IR, TGA, and SEM analyses. In vitro release studies demonstrated a sustained TMP release profile, best described by the Korsmeyer–Peppas diffusion model. Antibacterial assays confirmed the system’s effectiveness against *Staphylococcus aureus*, supporting its potential for localized and prolonged acne treatment. Moreover, cytocompatibility tests demonstrated that the formulation is biocompatible, further validating its suitability for topical application.

## 1. Introduction

The integration of host–guest chemistry and polymeric systems has led to the development of advanced drug delivery platforms, especially hydrogels and cryogels [1]. Among them, cyclodextrin-based gels represent a significant advancement due to their molecular recognition capabilities and biocompatibility. Cyclodextrins (CDs) are cyclic oligosaccharides capable of forming inclusion complexes with a wide range of guest molecules, enhancing the solubility, stability, and bioavailability of poorly water-soluble drugs [2]. γ-Cyclodextrin (γ-CD) is a cyclic oligosaccharide composed of eight α-1,4-linked D-glucopyranose units, resulting in a toroidal, hollow cone-shaped structure with a hydrophobic internal cavity and a hydrophilic outer surface due to the presence of hydroxyl groups [3]. This amphiphilic architecture allows γ-CD to form non-covalent inclusion complexes with a wide variety of guest molecules, particularly, large and poorly water-soluble (i.e., apolar) compounds. Notably, γ-CD has the largest internal cavity among natural cyclodextrins, with an approximate diameter of 0.95–1.0 nm, which significantly expands its complexation capacity compared to α- and β-cyclodextrins [4]. Due to this structural feature, γ-CD is frequently employed in pharmaceutical and material science applications to enhance the solubility, stability, and bioavailability of hydrophobic drugs and macromolecules, including peptides, lipophilic antibiotics, and even polymer chains [5,6]. Trimethoprim (TMP), a diaminopyrimidine antibiotic, is a broad-spectrum antimicrobial agent frequently employed in the management of acne, particularly in moderate-to-severe or treatment-resistant cases [7]. Acne vulgaris is a multifactorial skin condition affecting up to 85% of adolescents and a significant proportion of adults. It is primarily associated with sebum overproduction, follicular hyperkeratinization, and inflammation [8]. TMP is often used in combination with sulfamethoxazole or as a monotherapy in off-label dermatological applications [9]. However, its clinical use is limited by poor water solubility and instability in aqueous environments, particularly under light or oxidative stress, which affects its topical performance. In addition to its established use in dermatology, TMP has demonstrated significant antimicrobial activity against *Staphylococcus aureus*, including strains commonly implicated in acne exacerbations and secondary skin infections [10]. *S. aureus* colonization is frequently observed in inflammatory acne lesions and is associated with worsened clinical outcomes and increased resistance to conventional therapies [11]. Therefore, targeting *S. aureus* represents a valuable therapeutic strategy to enhance the overall efficacy of acne treatments, particularly in cases characterized by superinfection or antibiotic resistance.

γ-Cyclodextrin, due to its larger cavity size, is particularly suited for complexation with medium-sized molecules such as TMP [12,13]. Additionally, acrylated forms of cyclodextrins facilitate covalent incorporation into polymer matrices, such as hydrogels or cryogels, thereby enabling controlled drug release. Acrylate-based cryogels are widely used in drug delivery due to their tunable mechanical and swelling properties and their ability to incorporate bioactive agents [14,15]. Among the functional monomers employed in cryogel synthesis, [2-(acryloyloxy)ethyl]trimethylammonium chloride (AETMA) plays a distinctive role due to its permanent positive charge conferred by the quaternary ammonium group [16]. This cationic moiety renders the polymer electrostatically active, enabling strong interactions with negatively charged species such as bacterial cell membranes, anionic biomolecules, or contaminants. Moreover, AETMA has been shown to significantly enhance the adhesive properties of cryogels on biological surfaces, including skin, owing to its ability to form strong electrostatic interactions with the negatively charged components of the epidermis [17]. This feature is particularly advantageous for the development of topical drug delivery systems that require intimate and sustained contact with the skin [18].

In this work, we present the development of an innovative cryogel-based transdermal patch, engineered through the copolymerization of AETMA with an acrylated γ-cyclodextrin/trimethoprim (TMP) inclusion complex. This dual-functional system is specifically designed to synergistically enhance both the controlled release profile and the dermal retention of TMP, thereby maximizing its therapeutic potential. By ensuring prolonged skin contact and localized drug delivery, this cryogel patch holds significant promise as a next-generation treatment platform for acne therapy, addressing the need for effective, targeted, and patient-friendly dermatological solutions.

## 2. Results and Discussion

### 2.1. Synthesis of Monomer and Characterization

Inspired by previous findings demonstrating that native cyclodextrins can form stable inclusion complexes with poorly water-soluble drugs such as TMP, a strategy was developed to design a multifunctional cryogel system. To enable covalent incorporation into a hydrogel matrix, γ-CD was first functionalized through an acrylation reaction to yield acrylated γ-CD (γ-CDA), a polymerizable derivative bearing groups suitable for radical cryopolymerization [19].

The success of the acrylation process was confirmed via MALDI-TOF MS, which revealed a distribution of substitution products, with dominant peaks at *m*/*z* 1373, 1427, and 1481, corresponding to mono-, di-, and tri-acrylated γ-CD. The signal at *m/z* 1427 and 1481 confirmed the presence of a poly-acrylated species, enabling sufficient crosslinking during the cryogel formation [20].

**Figure 1:**
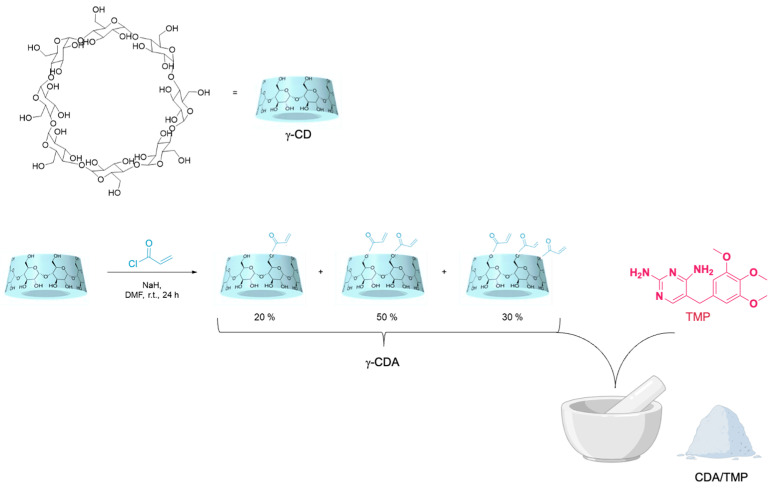
Schematic representation of the synthesis of γ-CD and CDA/TMP complex.

The γ-CDA/TMP complex was subsequently prepared via solvent-assisted kneading using a minimal amount of ethanol to facilitate the grinding process (Figure 1) [21].

In detail, equimolar amounts of the two components were ground together in a mortar for 45 min, then dried in vacuum for 24 h. The success of the co-grinding approach was confirmed through ^1^H NMR and thermal analysis, which demonstrated the effective integration of TMP into the cyclodextrin cavity.

**Figure 2:**
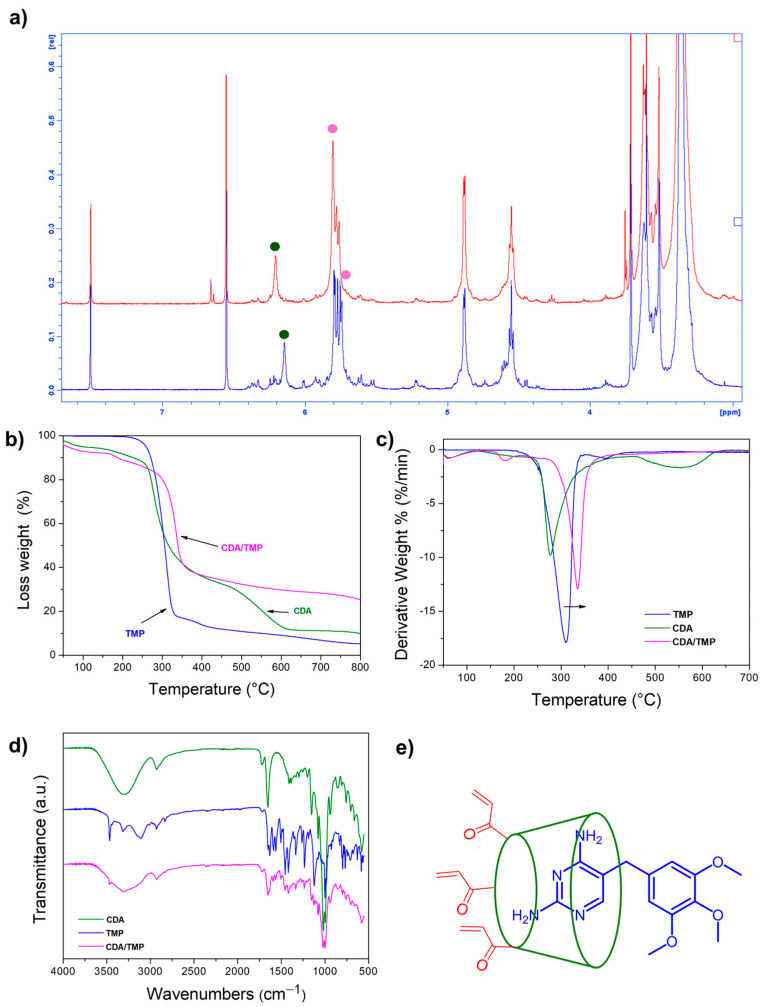
(**a**) ^1^H NMR of physical mixture (blue line) and CDA/TMP complex (red line); (**b**,**c**) TGA analysis; (**d**) ATR analysis of CDA and TMP alone and their inclusion complex (CDA/TMP); (**e**) schematic representation of the TMP region included in cyclodextrin cavity.

Upon comparison of the ^1^H NMR spectra of the physical mixture (CDA + TMP) and the CDA/TMP complex at the same concentration (Figure 2a), apparent differences were observed, indicating the effective formation of the host–guest complex. In particular, the aromatic protons corresponding to the –NH_2_ group of TMP exhibited downfield shifts upon complexation: The signal at 6.14 ppm in the physical mixture shifted to 6.20 ppm in the CDA/TMP complex, while the second –NH_2_ proton moved from 5.74 ppm to 5.80 ppm. These deshielding effects are consistent with the involvement of the amino group in host–guest interactions within the cyclodextrin cavity mediated by hydrogen bonds. Moreover, the signals corresponding to CDA protons in the complex appear broadened, which is consistent with the formation of an inclusion complex and suggests restricted mobility due to host–guest interactions.

Thermogravimetric analysis also confirmed the CDA/TMP complex formation. A comparison of the thermal behavior of the complex obtained, along with the related starting compounds, is shown in Figure 2b. In particular, CDA exhibits an initial weight loss due to its water content, followed by a degradation step at approximately 270 °C, with a residual mass at 800 °C of roughly 17%. This value of the residue, which is different in comparison to the value reported in the literature for γ-cyclodextrin, is likely due to structural distortion of the molecular ring caused by the reaction between the acrylic groups and the hydroxyl groups of γ-cyclodextrin [22]. TMP exhibits a sharp degradation event at approximately 310 °C, with a residual mass of less than 5% at 800 °C. On the other hand, the CDA/TMP complex displays a higher thermal degradation temperature compared to the individual components, as indicated by the shift in the derivative weight loss peak in Figure 2c. This thermal stabilization confirms the formation of the inclusion complex and suggests strong interactions between TMP and γ-CDA.

The inclusion complex CDA/TMP in the solid state was also investigated by ATR analysis. Figure 2e shows the FTIR spectra of TMP, CDA, and their inclusion complex (CDA/TMP). The spectrum of the complex does not display the emergence of new absorption bands, indicating the absence of covalent interactions. However, notable changes in intensity and slight shifts of characteristic peaks, particularly in the 1700–1000 cm^−1^ region, suggest successful formation of the inclusion complex [23]. These spectral modifications confirm interactions between TMP and the cyclodextrin cavity, consistent with host–guest complexation (Figure 2e).

### 2.2. Synthesis and Characterization of Cryogels

The two-step strategy, involving γ-cyclodextrin acrylation followed by complexation with TMP, facilitated the successful integration of the drug-loaded complex into the cryogel network, ensuring controlled release while maintaining structural compatibility.

To synthesize the cryogel designated as C_AETMA-CDA/TMP, the cationic co-monomer AETMA was combined with the acrylated γ-cyclodextrin monomer loaded with TMP (CDA/TMP). The integration of AETMA into the formulation confers enhanced electrostatic functionality and mechanical robustness, effectively overcoming the inherent structural limitations of cryogels composed entirely of cyclodextrin derivatives. The positively charged quaternary ammonium groups in AETMA not only contribute to the cryogel’s structural stability but also enable electrostatic interactions relevant for applications such as drug delivery and antimicrobial activity.

**Figure 3:**
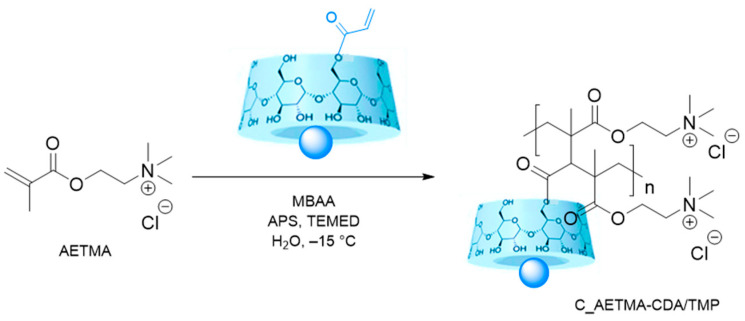
Schematic representation of C_AETMA-CD/TMP synthetic process.

A weight ratio of 1:2.5 (CDA/TMP to AETMA) was selected based on preliminary optimization studies, which demonstrated that this proportion provides a favorable balance among mechanical strength, swelling capacity, and overall gel stability. The co-polymerization reaction was carried out under cryogenic conditions, using water as the solvent, *N*,*N′*-methylenebis(acrylamide) (MBAA) as the crosslinking agent, and a standard radical initiation system. Polymerization at sub-zero temperature led to the formation of a macroporous cryogel network (Figure 3). Two control cryogels were synthesized under identical conditions for comparative purposes, one composed exclusively of AETMA (C_AETMA), and another based on a binary system of CDA and AETMA (C_CDA/AETMA), both without additional functional components.

**Figure 4:**
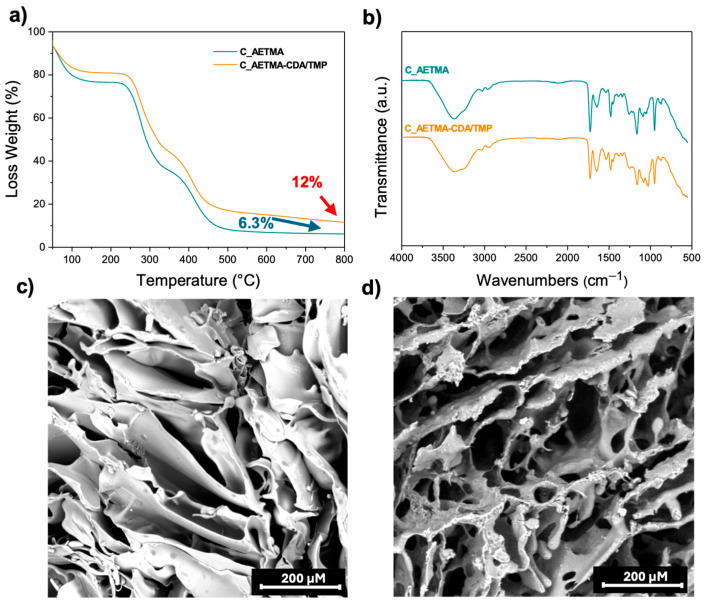
(**a**) Thermograms of C_AETMA and C_AETMA-CDA/TMP; (**b**) ATR of C_AETMA and C_AETMA-CDA/TMP; (**c**) SEM image of C_AETMA; (**d**) SEM image of C_AETMA-CDA/TMP.

Thermogravimetric analysis confirmed the thermal stability of the developed cryogels up to high temperatures, as shown in Figure 4a. Both samples exhibited a multistep degradation profile, including initial water loss and subsequent polymer decomposition. Notably, the C_AETMA-CDA/TMP sample retained a higher residual mass at 800 °C (~12%) compared to the C_AETMA (~6%), suggesting that the presence of CDA promotes the formation of more thermally resilient structures, likely through enhanced char formation [24]. These findings indicate that CDA not only contributes to the structural and functional architecture of the cryogel but also significantly enhances its thermal resistance.

Further confirmation of successful cryopolymerization is provided by the IR spectrum of the resulting material (Figure 4b). The cryogel displays characteristic signals confirming the occurrence of polymerization and the retention of functional groups from the monomers [25].

To gain insight into the cryogel morphology, scanning electron microscopy (SEM) was performed. The SEM images (Figure 4c,d) reveal a well-defined macroporous structure, characteristic of cryogels synthesized under sub-zero conditions. The network consists of interconnected polymer walls forming large, open pores ranging from 10 to 100 microns in diameter. This highly porous architecture, typical of cryotropic gelation, is expected to support efficient fluid uptake, nutrient diffusion, and the loading and gradual release of active substances, which are key features for biomedical and environmental applications. In some areas, discrete rounded features and surface irregularities were observed, possibly corresponding to polymer-rich domains formed during phase separation.

### 2.3. Swelling

**Figure 5:**
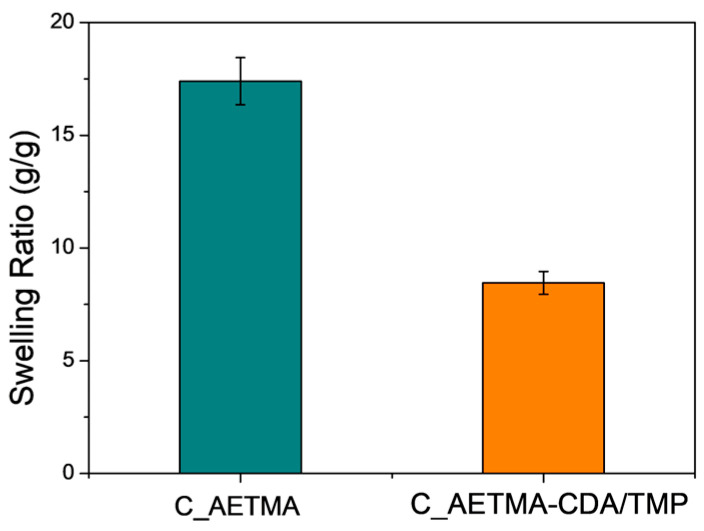
Swelling ratio of cryogels in PBS.

The swelling capacity of the C_AETMA-CDA/TMP was evaluated in phosphate-buffered saline (PBS, pH 7.4) to mimic physiological conditions. After 3 h of equilibration, the cryogel exhibited a swelling ratio of approximately 8.45 g/g, corresponding to an 845% increase in weight (Figure 5). This pronounced water uptake is primarily attributed to the cryogel’s highly interconnected macroporous structure, as well as to the hydrophilic nature of its components. CDA contributes numerous hydroxyl groups capable of forming hydrogen bonds with water, while the quaternary ammonium groups of AETMA facilitate electrostatic interactions with the surrounding medium. In contrast, C_AETMA displayed a significantly higher swelling ratio of 17.4 g/g, highlighting its intrinsic hydrophilicity. These results suggest that CDA contributes not only to drug complexation and stabilization but also to regulating the gel’s fluid absorption properties.

### 2.4. Drug Release Profile

**Figure 6:**
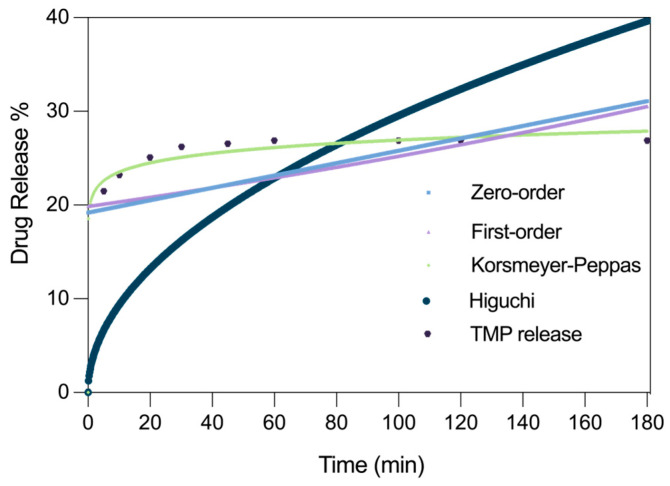
Drug release profile and fitting of TMP from C_AETMA-CDA/TMP.

To evaluate the drug release capacity of the C_AETMA-CDA/TMP system, in vitro release experiments were conducted, and the results were analyzed through UV spectroscopy. The release of TMP consisted of a two-step profile characterized by an initial rapid burst followed by a plateau phase. Within the first 5 min, approximately 21.4% of the loaded trimethoprim was released, increasing to ~26.2% by 30 min (Figure 6). This fast release phase is likely due to surface-associated drug molecules and loosely bound trimethoprim located near the outer pores of the cryogel, which are immediately accessible upon immersion in the release medium. Beyond 30 min, the release rate markedly decreased, and the profile entered a second phase, the plateau, where the release slowed significantly, gradually approaching a maximum cumulative release of ~26.84% after 180 min. The 180 min timepoint was selected as it encompassed the entire release profile, including the rapid initial phase and the subsequent plateau. No appreciable increase in drug release was detected beyond this timeframe, indicating that the system had reached a release equilibrium and that the accessible drug fraction had been thoroughly depleted under the experimental conditions. This biphasic release behavior is typical of supramolecular systems incorporating inclusion complexes (such as cyclodextrin-drug) within polymeric matrices. The release percentage at the plateau phase reflects that a fraction of the drug is still embedded within the polymeric matrix, primarily in the form of a stable inclusion complex with cyclodextrin. This molecular arrangement effectively prevents early and uncontrolled release into the aqueous environment. The overall limited release is consistent with the high stability of the cyclodextrin–TMP complex in aqueous environments, supporting the observed retention.

Nonlinear regression fitting was performed using several kinetic models, including zero-order, first-order, Higuchi, Korsmeyer–Peppas, and Weibull. Among these, the Korsmeyer–Peppas model provided the best fit, with an R^2^ value of 0.99, indicating excellent agreement with the experimental data (Table 1). This suggests a diffusion-controlled release mechanism, possibly involving Fickian diffusion [26].

The zero- and first-order models did not adequately capture the initial burst release, resulting in lower correlation coefficients. The Higuchi model, although suitable for systems governed purely by diffusion, underestimated the plateau phase and failed to account for the full release behavior. Attempts to apply the Weibull model were unsuccessful. The model failed to converge or provided poor fits, likely due to the very sharp initial release followed by an early plateau. The Weibull function requires a more gradual sigmoidal release profile and is not well suited to systems exhibiting an immediate burst followed by minimal change.

### 2.5. Antimicrobial Activity

The results of the antimicrobial assay are summarized in Table 2. The standard trimethoprim disk yielded an average inhibition zone of 16.2 mm, confirming its expected antibacterial efficacy. In contrast, unloaded hydrogels C_AETMA and C_AETMA-CDA did not exhibit any measurable inhibition zones, indicating the absence of intrinsic antimicrobial activity by the polymeric matrices alone.

Notably, the formulation C_AETMA-CDA/TMP demonstrated a markedly larger inhibition zone of 25.2 mm, significantly surpassing that of the pure antibiotic disk. This enhanced activity suggests that the cryogel matrix not only retains the drug’s efficacy but may potentiate it, potentially through improved diffusion or sustained release mechanisms. The CD component appears to play an essential role by enhancing the solubility and local concentration of trimethoprim near the diffusion front, a phenomenon previously observed in drug-delivery systems employing CD-based carriers [27,28].

These results indicate that the C_AETMA-CDA/TMP system functions as an efficient drug delivery vehicle, enhancing the bioavailability and antimicrobial efficacy of trimethoprim against *S. aureus*. The increase in zone size suggests either a more efficient diffusion gradient or prolonged release of the active molecule compared to the pure form applied on a paper disk. Similar trends have been reported for hydrogel-embedded antibiotics, where matrix composition and porosity influence drug diffusion and antimicrobial efficacy [29,30].

Furthermore, the lack of an antimicrobial effect in the absence of the antibiotic confirms that the observed activity is drug-dependent, demonstrating the clinical utility of such formulations in local infection treatment, particularly where bioavailability and tissue retention of the drug are critical.

### 2.6. Cytotoxic Activity

**Figure 7:**
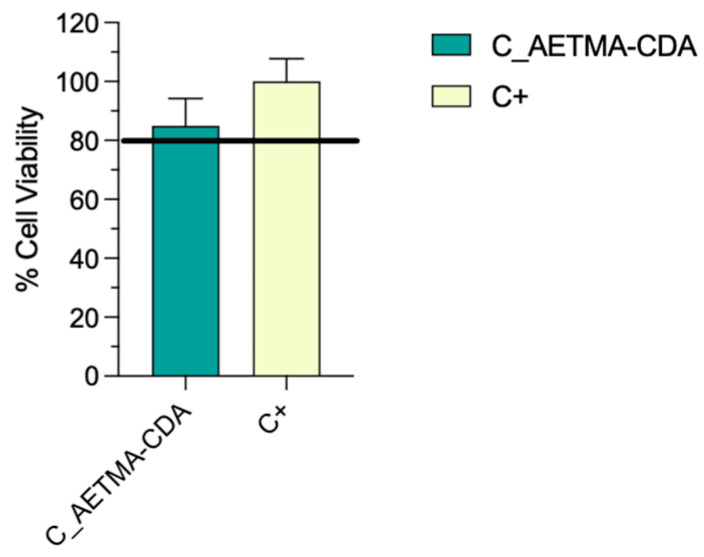
Evaluation of cell viability in HCT-116 cells after 24 h exposure to disks using the MTT assay. Cell viability is expressed as a percentage relative to the untreated control (C+). Data are shown as mean ± standard deviation (n = 3). Statistical analysis was performed using an unpaired two-tailed Student’s *t*-test. The resulting *p*-value was 0.8280, indicating no statistically significant difference between treated and control groups (*p* > 0.05).

C_AETMA-CDA/TMP did not induce significant cytotoxic effects on HCT-116 cells after 24 h of treatment. As shown in Figure 7, the average cell viability in the treated group was 85%. Since this value exceeded the pre-established cytotoxicity threshold of 80%, the material can be considered non-cytotoxic under the tested conditions. These findings suggested that the disks are biocompatible with HCT-116 cells for 24 h exposure periods. Cell viability consistently remained above the critical cytotoxicity threshold, indicating low adverse effects on cellular metabolism and survival. Further investigations will be necessary to assess long-term exposure, varying concentrations, and the impact on other cell types.

## 3. Materials and Methods

### 3.1. Materials

Trimethoprim (TMP), γ-CD, acryloyl chloride, sodium hydride in mineral oil (60%), acetone, dimethylformamide anhydrous (DMF), ethanol, *N*,*N′*-methylene-bisacrylamide (MBAA), [2-(Acryloyloxy)ethyl]trimethylammonium chloride solution (80 wt.% in H_2_O) (AETMA), ammonium persulfate (APS), and *N*,*N*,*N′*,*N′*-tetramethylethylenediamine (TEMED) were purchased from Sigma-Aldrich (Merck Life Science S.r.l., Milan, Italy). A Milli-Q water purification system was used to produce deionized water. ^1^H NMR spectra were recorded at 300 K on Varian UNITY Inova 400 MHz using DMSO-*d_6_* as solvent.

### 3.2. Chemistry

#### 3.2.1. Synthesis of γ-CD-Acrylate Monomers (CDA)

γ-CD-acrylate monomers were synthesized according to previously published literature [20]. In detail, γ-CD (0.5 g, 0.385 mmol) was dissolved in DMF (7 mL) and cooled to 0 °C. Under nitrogen flux, NaH (61.67 mg, 1.541 mmol, 4 eq) was added, and the mixture was stirred for 1 h. Then, acryloyl chloride (128.6 µL, 1.541 mmol, 4 eq) was added dropwise, and the mixture was allowed to stir for 24 h at room temperature. At the end of the reaction, cold acetone was added, forming a white solid that was collected by filtration and dried in an oven for 24 h. Yield = 70%. MALDI–TOF *m*/*z* = 1319, 1373, 1427.

#### 3.2.2. Synthesis of γ-CD–Acrylate–Trimethoprim Inclusion Complex (CDA/TMP)

The CDA/TMP inclusion complex was prepared using a kneading method. Based on the average molar mass of CDA (1.4 kg/mol), 1.4 kg of CDA (1.0 mmol) and 290 mg of TMP (1.0 mmol) were weighed and transferred into a mortar [31]. A few microliters of ethanol were gradually added dropwise to moisten the mixture, forming a paste, and the kneading process was carried out for 45 min under manual pressure to facilitate complexation. The obtained mixture was dried at 40 °C under vacuum. The obtained product was pulverized to obtain a uniform white powder, characterized by TGA, DSC, and FT-IR analysis.

#### 3.2.3. Synthesis of CDA/TMP-Based Cryogel (C_AETMA-CDA/TMP)

A cryogel incorporating the CDA/TMP inclusion complex was prepared via free radical polymerization conducted at sub-zero temperature. Specifically, a mixture of the CDA/TMP complex and [2-(acryloyloxy)ethyl]trimethylammonium chloride (AETMA), used as a co-monomer, was solubilized in distilled water at a 1:2.5 weight ratio. As a crosslinking agent, MBAA was introduced at a 6:1 molar ratio relative to the total monomer content. The volume was then adjusted to reach a final concentration of 10% *w*/*v* polymerizable material in solution. Subsequently, 2% *v*/*v* of an aqueous solution of ammonium persulfate (APS, 10% *w*/*v*) was added as the initiator, followed by 2% *v*/*v* of an aqueous TEMED (10% *w*/*v*) solution to catalyze polymerization. The resulting mixture was stirred briefly (~1 min) and then transferred into a petri dish. The system was left to polymerize in a freezer for 72 h. After gelation, the resulting cryogel was thoroughly washed with distilled water and ethanol to remove unreacted components and then subjected to drying under a nitrogen stream, followed by overnight vacuum drying at 40 °C.

A control material (blank cryogel), referred to as C_AETMA-CDA, was synthesized under identical conditions using CDA and AETMA without TMP to evaluate the contribution of the drug to the cryogel’s structural and functional properties. Additionally, a second control cryogel composed solely of AETMA (C_AETMA) was prepared to assess the role of the cyclodextrin component.

### 3.3. Characterization

#### 3.3.1. MALDI-TOF MS

The acrylation of γ-cyclodextrin was confirmed by MALDI-TOF MS (analysis using a Bruker Ultraflex apparatus (Bruker Daltonics, Billerica, MA, USA) operating in reflective mode, with 2,5-dihydroxybenzoic acid (DHB) as the matrix in positive ion mode. The spectra displayed multiple peaks consistent with the gradual acrylation of γ-cyclodextrin. The detected sodium adducts [M+Na]^+^ showed signals at

*m*/*z* 1373, consistent with monoacrylated γ-CD;
*m*/*z* 1427, assigned to diacrylated γ-CD;
*m*/*z* 1481, corresponding to triacrylated γ-CD.

These findings confirm partial substitution of γ-cyclodextrin with 1 to 3 acrylate groups, with a molecular mass shift of approximately 54 Da per acrylate unit.

#### 3.3.2. Infrared Spectroscopy (FT-IR)

FTIR-ATR analysis was carried out using a Thermo Nicolet IS50 (Thermo Fisher Scientific, Waltham, MA, USA) with an integrated diamond ATR sampling module. The spectra were collected over 64 scans in the 4000–500 cm^−1^ range, with a resolution of 4 cm^−1^, at room temperature.

#### 3.3.3. Thermogravimetric Analysis (TGA)

Thermogravimetric analysis (Perkin Elmer Inc., Waltham, MA, USA) was performed using a TA Instruments Q500 apparatus under a nitrogen atmosphere (flow rate: 60 mL/min) with a heating rate of 10 °C/min over a temperature range of 50 °C to 800 °C. The TGA system features a sensitivity of 0.1 μg and a weighing precision of ±0.01%, with an isothermal temperature accuracy of ±1 °C.

#### 3.3.4. Cryogel’s Morphologies

Cryogel’s morphologies were analyzed using SEM. Characterization was performed using a Thermo Phenom ProX desktop SEM (Thermo Fisher Scientific, Waltham, MA, USA).

### 3.4. Swelling Measurement

The water absorption capacity of the cryogel was evaluated under physiological-like conditions using phosphate-buffered saline (PBS, pH 7.4). Initially, the cryogel samples were oven-dried at 50 °C until they reached a constant weight. The dry weight (*W*_0_) of each sample was measured using an analytical balance with a precision of 0.01 mg. Subsequently, the dried hydrogels were immersed in PBS at room temperature for 3 h to allow swelling. After incubation, samples were gently removed and blotted with filter paper to eliminate surface liquid without altering the structure. The swollen weight (*W_s_*) was then immediately recorded.

The swelling ratio (*SR*) was calculated using the following equation:

(1) $$SR=\frac{(W_{s}-W_{0})\;}{W_{0}}$$

All experiments were performed in triplicate, and the results are presented as mean values with standard deviation.

### 3.5. Drug Release Experiments

The drug release behavior of TMP from the cryogel matrix was investigated under simulated physiological conditions using phosphate-buffered saline (PBS, pH 7.4) as release medium. Accurately weighed samples of cryogel (2 mg) containing the CDA/TMP complex were immersed in 2 mL of PBS and maintained under gentle agitation using an orbital shaker at room temperature.

At predetermined time intervals (0, 5, 10, 20, 30, 45, 60, 80, 100, 120, and 180 min), aliquots of the release medium (20 μL), were withdrawn and immediately analyzed via UV–Vis spectrophotometry by monitoring the absorbance at 209 and 278 nm, the characteristic absorption maximum for trimethoprim. To preserve sink conditions, each sampled volume was replaced with an equal volume of fresh PBS.

The concentration of released TMP in each aliquot was determined based on a previously established calibration curve (R^2^ > 0.99), and the cumulative release percentage was calculated accordingly.

All release experiments were carried out in triplicate, and the data are reported as mean ± standard deviation.

Kinetic modeling of the release profiles was performed using GraphPad Prism 9 software. Curve fitting was carried out according to standard release models, including zero-order, first-order, Higuchi, Korsmeyer–Peppas, and Weibull models, using the corresponding equations as reported in literature [32].

### 3.6. Antimicrobial Assay

The antibacterial activity of the tested materials was evaluated against *S. aureus* ATCC 25213 using the agar disk diffusion method. Bacterial strain was obtained from the American Type Culture Collection (ATCC, Manassas, VA, USA). Test samples, consisting of different hydrogel formulations, were applied as disks onto Luria Bertani agar plates (LB Base Lennox L-2897 Sigma-Aldrich) previously inoculated with the bacterial suspension at a concentration of 1.5 × 10^8^ CFU/mL. A commercial 5 µg trimethoprim disk (Thermo Scientific^TM^ Oxoid^TM^ CT0076B, Rodano (MI), Italy) was used as a positive control. After aerobic incubation at 37 °C overnight, inhibition zones were measured using a caliper and expressed in millimeters.

### 3.7. Cytotoxic Activity

A cytotoxicity assay was performed using the MTT test on human colorectal carcinoma cells HCT-116 (ATCC^®^ CCL-247^TM^). Cell lines were obtained from the American Type Culture Collection (ATCC, Manassas, VA, USA). HCT-116 cells were cultured in RPMI 1640 (PanBiotek P04-18500, Aidenbach, Germany) supplemented with 2 mM l-glutamine, 10% fetal bovine serum (FBS), and antibiotics (100 U/mL penicillin and 100 μg/mL streptomycin). Briefly, as previously described [19], cells were seeded at a density of 1.5 × 10^5^ cells per well in 24-well plates and incubated under standard conditions at 37 °C and 5% CO_2_ until reaching approximately 80% confluence. At this point, the disks were inserted into each well. Wells containing only the cells served as an untreated control group.

After 24 h of incubation with the disks, the MTT assay was performed to assess cell viability. MTT powder (3-(4,5-dimethylthiazol-2-yl)-2,5-diphenyltetrazolium bromide) was dissolved in serum-free medium at a final concentration of 0.5 mg/mL. Then, the cells were gently washed with PBS, and 500 μL of the MTT solution was added to each well. The plate was then incubated for 3 h at 37 °C. Formazan crystals were dissolved using 500 μL of DMSO and then quantified using a Biotek Synergy^TM^ HTX spectrophotometer at 570 nm.

The experimental condition was tested in triplicate to ensure reproducibility. Absorbance values were normalized to the untreated control group, and cell viability was expressed as a percentage relative to this control. A cytotoxicity threshold was defined at 80% viability, following ISO 10993-5 guidelines for the biological evaluation of medical devices [33].

### 3.8. Statistical Analysis

All data were analyzed using GraphPad Prism version 10 (GraphPad Software, San Diego, CA, USA). Cytotoxicity results obtained from the MTT assay were expressed as mean ± standard deviation (SD) of three independent replicates. To compare the viability of treated versus control groups, an unpaired two-tailed Student’s t-test was employed. Before the analysis, data normality was assessed using the Shapiro–Wilk test to ensure the appropriateness of parametric testing. A *p*-value <0.05 was considered statistically significant.

## 4. Conclusions

In this study, a novel macroporous material with bioadhesive properties, a monomer, and drug delivery performance was successfully synthesized and fully characterized. By taking advantage of the γ-cyclodextrin embedded within the material scaffold, the system demonstrated the ability to protect and improve the bioavailability of trimethoprim through the formation of a supramolecular inclusion complex. This interaction not only stabilizes the active pharmaceutical ingredient but also enables controlled release of the drug. Furthermore, the bioadhesive functionality, derived from the incorporation of the AETMA moiety into the polymeric framework, contributes to prolonged retention on the skin surface, thereby enhancing local drug residence time. Overall, the synergistic combination of AETMA’s adhesive properties and γ-cyclodextrin’s host–guest complexation offers a promising approach for the development of advanced drug delivery platforms, particularly for topical administration.

## Figures and Tables

**Table 1:** Kinetic release parameters for TMP from cryogel.

Model	*R^2^*	*k*	*n*
Zero-order	0.2220	−0.06605	—
First-order	0.1956	−0.002385	—
Korsmeyer–Peppas	0.9915	20.54	0.05889
Higuchi	−0.4793	2.954	—

**Table 2:** Results of the agar diffusion assay. Inhibition zones were expressed as mean values (mm) ± 0.1.

Sample	Inhibition Zone (mm)
C_AETMA-CDA/TMP	25.2
C_AETMA	NA
C_AETMA-CDA	NA
TMP (standard disk)	16.2

## Data Availability

The original contributions presented in this study are included in the article. Further inquiries can be directed to the corresponding author.

## References

[1] Omidian H., Akhzarmehr A., Gill E.J. (2025). Cyclodextrin–Hydrogel Hybrids in Advanced Drug Delivery. Gels.

[2] Crini G. (2014). Review: A History of Cyclodextrins. Chem. Rev..

[3] Wüpper S., Lüersen K., Rimbach G. (2021). Cyclodextrins, Natural Compounds, and Plant Bioactives—A Nutritional Perspective. Biomolecules.

[4] Loftsson T., Duchêne D. (2007). Cyclodextrins and Their Pharmaceutical Applications. Int. J. Pharm..

[5] Chaudhari P., Ghate V.M., Lewis S.A. (2019). Supramolecular Cyclodextrin Complex: Diversity, Safety, and Applications in Ocular Therapeutics. Exp. Eye Res..

[6] Astray G., Gonzalez-Barreiro C., Mejuto J.C., Rial-Otero R., Simal-Gándara J. (2009). A Review on the Use of Cyclodextrins in Foods. Food Hydrocoll..

[7] Bottomley W.W., Cunliffe W.J. (2009). Oral Trimethoprim as a Third-Line Antibiotic in the Management of Acne Vulgaris. Dermatology.

[8] Zaenglein A.L., Pathy A.L., Schlosser B.J., Alikhan A., Baldwin H.E., Berson D.S., Bowe W.P., Graber E.M., Harper J.C., Kang S. (2016). Guidelines of Care for the Management of Acne Vulgaris. J. Am. Acad. Dermatol..

[9] McCarty M., Rosso J.Q.D. (2011). Chronic Administration of Oral Trimethoprim-Sulfamethoxazole for Acne Vulgaris. J. Clin. Aesthet. Dermatol..

[10] DEL GIUDICE P. (2020). Skin Infections Caused by Staphylococcus Aureus. Acta Derm. Venereol..

[11] Khorvash F., Abdi F., Kashani H.H., Naeini F.F., Narimani T. (2012). Staphylococcus Aureus in Acne Pathogenesis: A Case-Control Study. N. Am. J. Med. Sci..

[12] Arti S., Kaur K., Kaur J., Ghosh T.K., Banipal T.S., Banipal P.K. (2021). Host-Guest Interaction of Trimethoprim Drug with Cyclodextrins in Aqueous Solutions: Calorimetric, Spectroscopic, Volumetric and Theoretical Approach. J. Mol. Liq..

[13] Oliveri V., D’Agata R., Giglio V., Spoto G., Vecchio G. (2013). Cyclodextrin-Functionalised Gold Nanoparticles via Streptavidin: A Supramolecular Approach. Supramol. Chem..

[14] Zagni C., Patamia V., Dattilo S., Fuochi V., Furnari S., Furneri P.M., Carroccio S.C., Floresta G., Rescifina A. (2024). Supramolecular Biomaterials as Drug Nanocontainers with Iron Depletion Properties for Antimicrobial Applications. Mater. Adv..

[15] Omidian H., Dey Chowdhury S., Babanejad N. (2023). Cryogels: Advancing Biomaterials for Transformative Biomedical Applications. Pharmaceutics.

[16] Rodrigues A.S., Charreyre M.-T., Favier A., Baleizão C., Farinha J.P.S. (2019). Temperature-Responsive Copolymers without Compositional Drift by RAFT Copolymerization of 2-(Acryloyloxy)Ethyl Trimethylammonium Chloride and 2-(Diethylamino)Ethyl Acrylate. Polym. Chem..

[17] Jiang Z., Li Y., Shen Y., Yang J., Zhang Z., You Y., Lv Z., Yao L. (2021). Robust Hydrogel Adhesive with Dual Hydrogen Bond Networks. Molecules.

[18] Zhang R., Liberski A., Sanchez-Martin R., Bradley M. (2009). Microarrays of over 2000 Hydrogels—Identification of Substrates for Cellular Trapping and Thermally Triggered Release. Biomaterials.

[19] Tomarchio E.G., Zagni C., Dattilo S., Vitiello L., Fuochi V., Furnari S., Furneri P.M., Granata G., Carroccio S.C., Rescifina A. (2025). Advanced Cyclodextrin-Based Multiloaded Hydrogels for Targeted Drug Delivery in the Fight against Vaginal Fungal Infections. Carbohydr. Polym..

[20] Zagni C., Coco A., Mecca T., Curcuruto G., Patamia V., Mangano K., Rescifina A., Carroccio S.C. (2023). Sponge-like Macroporous Cyclodextrin-Based Cryogels for Controlled Drug Delivery. Mater. Chem. Front..

[21] Jug M., Mura P.A. (2018). Grinding as Solvent-Free Green Chemistry Approach for Cyclodextrin Inclusion Complex Preparation in the Solid State. Pharmaceutics.

[22] Spitaleri F., Dattilo S., Aleo D., Saita M.G., Patti A. (2025). Cyclodextrin-Based Iodophors with High Iodine Retention in Solid State and in Dilute Solutions. Carbohydr. Polym..

[23] Ding Y., Ma Y., Ding C., Nie J., Xu Z. (2025). Preparation of Trimethoprim/Methyl *β*-Cyclodextrin Complexes, In Vitro and In Vivo Pharmacokinetic Study, and Evaluation of Antibacterial Activity in Combination with the Complex of Sulfamethoxazole/Methyl *β*-Cyclodextrin. J. Mol. Struct..

[24] Dattilo S., Spitaleri F., Aleo D., Saita M.G., Patti A. (2023). Solid-State Preparation and Characterization of 2-Hydroxypropylcyclodextrins-Iodine Complexes as Stable Iodophors. Biomolecules.

[25] Dattilo S., Zagni C., Mecca T., Patamia V., Floresta G., Nicotra P., Carroccio S.C., Rescifina A. (2024). Solvent-Free Conversion of CO_2_ in Carbonates through a Sustainable Macroporous Catalyst. Giant.

[26] Ferrero C., Massuelle D., Doelker E. (2010). Towards Elucidation of the Drug Release Mechanism from Compressed Hydrophilic Matrices Made of Cellulose Ethers. II. Evaluation of a Possible Swelling-Controlled Drug Release Mechanism Using Dimensionless Analysis. J. Control Release.

[27] Loftsson T., Brewster M.E. (2010). Pharmaceutical Applications of Cyclodextrins: Basic Science and Product Development. J. Pharm. Pharmacol..

[28] Szente L., Szejtli J. (2004). Cyclodextrins as Food Ingredients. Trends Food Sci. Technol..

[29] Hoare T.R., Kohane D.S. (2008). Hydrogels in Drug Delivery: Progress and Challenges. Polymer.

[30] Qiu Y., Park K. (2012). Environment-Sensitive Hydrogels for Drug Delivery. Adv. Drug Deliv. Rev..

[31] Jablan J., Bačić I., Kujundžić N., Jug M. (2013). Zaleplon Co-Ground Complexes with Natural and Polymeric β-Cyclodextrin. J. Incl. Phenom. Macrocycl. Chem..

[32] Zagni C., Scamporrino A.A., Riccobene P.M., Floresta G., Patamia V., Rescifina A., Carroccio S.C. (2023). Portable Nanocomposite System for Wound Healing in Space. Nanomaterials.

[33] (2009). Biological Evaluation of Medical Devices.

